# Underweight, overweight, and obesity as independent risk factors for hospitalization in adults and children from influenza and other respiratory viruses

**DOI:** 10.1111/irv.12618

**Published:** 2018-12-04

**Authors:** Joe‐Ann S. Moser, Arturo Galindo‐Fraga, Ana A. Ortiz‐Hernández, Wenjuan Gu, Sally Hunsberger, Juan‐Francisco Galán‐Herrera, María Lourdes Guerrero, Guillermo M. Ruiz‐Palacios, John H. Beigel, The La Red ILI 002 Study Group, Martin Magaña‐Aquino, Rafael Valdez‐Vazquez, Sarbelio Moreno‐Espinosa, Arturo Galindo‐Fraga, Alejandra Ramírez‐Venegas, Beatriz Llamosas‐Gallardo, Santiago Pérez‐Patrigeon, Daniel Ernesto Noyola Cherpitel

**Affiliations:** ^1^ National Institute of Allergy and Infectious Diseases Bethesda Maryland; ^2^ Instituto Nacional de Ciencias Médicas y Nutrición Salvador Zubirán Mexico City Mexico; ^3^ Instituto Nacional de Pediatría Mexico City Mexico; ^4^ Leidos Biomedical Research Inc. in support of National Institute of Allergy and Infectious Diseases Bethesda Maryland; ^5^ Mexico Emerging Infectious Disease Clinical Research Network Coordinating Center Mexico City Mexico; ^6^ Comisión Coordinadora de Institutos Nacionales de Salud y Hospitales de Alta Especialidad Mexico City Mexico

**Keywords:** body mass index, hospital burden of disease, influenza, obesity, respiratory viral pathogens

## Abstract

**Background:**

The relationship between obesity and risk of complications described during the 2009 influenza pandemic is poorly defined for seasonal influenza and other viral causes of influenza‐like illness (ILI).

**Methods:**

An observational cohort of hospitalized and outpatient participants with ILI was conducted in six hospitals in Mexico. Nasopharyngeal swabs were tested for influenza and other common respiratory pathogens.

**Results:**

A total of 4778 participants were enrolled in this study and had complete data. A total of 2053 (43.0%) had severe ILI. Seven hundred and seventy‐eight (16.3%) were positive for influenza, 2636 (55.2%) were positive for other viral respiratory pathogens, and 1364 (28.5%) had no respiratory virus isolated. Adults with influenza were more likely to be hospitalized if they were underweight (OR: 5.20), obese (OR: 3.18), or morbidly obese (OR: 18.40) compared to normal‐weight adults. Obese adults with H1N1 had a sixfold increase in odds of hospitalization over H3N2 and B (obese OR: 8.96 vs 1.35, morbidly obese OR: 35.13 vs 5.58, respectively) compared to normal‐weight adults. In adults with coronavirus, metapneumovirus, parainfluenza, and rhinovirus, participants that were underweight (OR: 4.07) and morbidly obese (OR: 2.78) were more likely to be hospitalized as compared to normal‐weight adults. All‐cause influenza‐like illness had a similar but less pronounced association between underweight or morbidly obesity and hospitalization.

**Conclusions:**

There is an increased risk of being hospitalized in adult participants that are underweight or morbidly obese, regardless of their viral pathogen status. Having influenza, however, significantly increases the odds of hospitalization in those who are underweight or morbidly obese.

## 1 BACKGROUND

Until the onset of the 2009 influenza A/H1N1 pandemic, body mass index (BMI) was not widely appreciated as an independent risk factor for influenza. While there was no increase in the rates of ILI being reported in obese patients,^1^ early reports during the pandemic noted an association between severity of illness and obesity.^2^, ^3^, ^4^, ^5^, ^6^, ^7^, ^8^, ^9^, ^10^, ^11^ Several studies in adults found an association between obesity and death due to pandemic influenza.^11^, ^12^, ^13^, ^14^ Another study, however, found no association between obesity and mortality in intensive care unit (ICU) participants.^15^

Far fewer studies examined syndromic influenza‐like illness (rather than restricted to influenza). Two studies found a link between obesity and hospitalization from influenza‐like illness,^16^, ^17^ and another showed higher rates of outpatient visits for influenza‐like illness in obese patients.^18^ One additional study evaluated rates of obesity in the community and demonstrated that communities with a greater prevalence of obesity were more likely to have high influenza‐related hospitalization rates.^19^ None of these studies, however, determined the etiology of the participants illness, and none examined low body mass index as a risk factor.

Given the limited data with syndromic ILI severity and obesity, we examined the association between body mass and severity of influenza‐like illness (ILI) during four consecutive years (2010‐2014) in both pediatric and adult populations. We hypothesized that those individuals categorized as overweight, obese, and morbidly obese would be more likely to be hospitalized with ILI from influenza, but also from other respiratory viruses, as compared to those participants with a normal body mass.

## 2 METHODS

### 2.1 Study design and sites

Beginning in April 2010, participants were enrolled in ILI 002, an observational cohort study conducted by the Mexican Emerging Infectious Disease Clinical Research Network, Mexico (La Red). The study was conducted at five centers in Mexico City, Mexico, and one in San Luis Potosí, Mexico. The study sites are located in urban environments and include two general hospitals, two tertiary care hospitals (one that serves those with respiratory problems and one that serves those with metabolic disorders), and two tertiary care pediatric centers.

### 2.2 Case definition and study population

The study population included participants of any age who presented with an influenza‐like illness (ILI). Influenza‐like illness was defined by the presence of at least one respiratory symptom (eg, shortness of breath, cough) and either fever (≥38°C or subjective feverishness) or one or more non‐respiratory symptoms (eg, malaise, headache). The participants included were those who sought medical attention at a study center and agreed to participate in the study.

### 2.3 Study procedures

At enrollment, demographic data were collected; height, weight, and vital signs were measured; and the presence of chronic medical conditions (congenital malformation/congenital syndrome, cardiovascular disorder, chronic pulmonary disease, chronic obstructive pulmonary disease, asthma, liver disorder, renal disorder, diabetes mellitus, immunodeficiency, etc.) was recorded. A nasopharyngeal swab (Copan, Brescia, Italy), or a nasal aspirate, was obtained for PCR detection of respiratory pathogens. Follow‐up information (symptoms, impact on daily function, hospitalizations, and death) was obtained on days 14 and 28.

### 2.4 Virology

Nasopharyngeal swab and nasal aspirate samples were sent to a central laboratory (Molecular Biology Laboratory, Infectious Diseases Department, INCMNSZ, Mexico City) and stored at −70°C. All samples were tested by real‐time reverse transcription‐PCR for influenza A following the Centers for Disease Control and Prevention protocol.^20^ All samples were also tested for multiple pathogens with either the RespiFinder 19 (from April 2010 to May 2012) or RespiFinder 22 (from June 2012 to March 2014) kit (PathoFinder B.V., Maastricht, the Netherlands).^21^ The 19‐plex PCR test can detect and differentiate between 15 viruses (coronavirus NL63, OC43, and 229E; human metapneumovirus, influenza A, influenza A H5N1, influenza B, parainfluenza virus types 1‐4; respiratory syncytial virus types A and B; rhinovirus; and adenovirus) and four bacteria (*Bordetella pertussis*,* Chlamydophila pneumoniae*,* Legionella pneumophila*, and *Mycoplasma pneumoniae*). The 22‐plex test added coronavirus HKU1, bocavirus, enterovirus, and influenza A/H1N1 pdm 2009 while removing influenza A H5N1. The analytical sensitivity of the assay varies between 5 and 50 copies per reaction for most targets.^22^

### 2.5 Subject classification

For adults (age ≥ 19 years), body mass index (BMI) was calculated using weight(kg)/[height (m)^2^] and the World Health Organization (WHO) International Classification: underweight (BMI < 18.5), normal (18.5 ≤ BMI < 25.0), overweight (25.0 ≤ BMI < 30.0), obese (30.0 ≤ BMI < 35.0), and morbidly obese (BMI ≥ 35.0). For children and adolescents aged < 19 years (collectively called “pediatric participants”), WHO Child Growth Standards were used. We calculated a z‐score for each pediatric participant in the study and assigned a body mass index category as follows: underweight (*z*‐score < −1.0), normal (−1.0 ≤ *z*‐score ≤ 1.0), overweight (1.0 < *z*‐score ≤ 2.0), obese (2.0 < *z*‐score ≤ 3.0), and morbidly obese (>3.0).

Participants were classified according to three viral pathogen groupings using the following definitions: The first group included influenza‐positive participants who were confirmed positive for only influenza A or B, or co‐infected with influenza and another pathogen; the second group is composed of non‐influenza respiratory virus‐positive (NIRV positive) participants who were negative for influenza but positive for one or more of the five most commonly isolated non‐influenza respiratory viruses (rhinovirus/enterovirus, coronavirus, respiratory syncytial virus (RSV), parainfluenza virus (PIV), and metapneumovirus); and the third group included individuals that had either no pathogen or only bacterial pathogens isolated from their samples (virus negative).

The outcome measure of severity of disease was defined as having severe disease if participants were hospitalized within 14 days of enrollment. Participants that were in the emergency room >24 hours yet never admitted were excluded as these cannot be clearly categorized as outpatients or hospitalized.

### 2.6 Statistical analyses

All analyses were performed separately for pediatric and adult participants. Descriptive statistics include presentation of proportion for categorical variables and means and standard deviations for continuous variables. Logistic regression models were used to examine the relationship between severity of illness and BMI. Sex, chronic medical condition (present/absent), and age (as a linear term) were included in all models to adjust for imbalances.

Interactions between BMI and other covariates (adjustment variables and pathogen categories) were examined. When interaction terms were significant, logistic regression analyses were performed for each level of the covariate. We calculated the odds ratio (OR) of being hospitalized based on body mass category, where normal was the reference category. We estimated 95% confidence intervals (95% CI) and *P*‐values of the odds ratios using the Firth method to account for separation that may occur in logistic regression models due to small sample sizes.^23^ SAS version 9 (SAS Institute Inc., Cary, NC, USA) and R 3.1.0 (R Foundation for Statistical Computing, Vienna, Austria) were used to complete all analysis.

### 2.7 Regulatory aspects

The Institutional Review Board (IRB) at each site approved this study. The study was conducted following the principles of the International Conference on Harmonization's Good Clinical Practice, Declaration of Helsinki, and the Mexican General Health Law. All participants provided informed consent. The project was registered on clinicaltrials.gov (NCT01418287).

## 3 RESULTS

Between April 2010 and March 2014, 5678 participants were enrolled. Participants were excluded from this analysis for the following reasons: 33 were pregnant, 233 had missing height and/or weight data, 602 remained in the emergency room >24 hours yet were not admitted to the hospital, and 32 had missing pathogen information. Of the remaining 4778 participants, 1530 (32.0%) were pediatric participants (under the age of 19 years). Nine hundred and seventy (63.4%) of the pediatric participants and 1083 (33.3%) of adult participants were hospitalized (Table 1). In the pediatric cohort, those hospitalized were much younger than those that did not require hospitalization (average age of 2.44 years vs 6.32 years, respectively). The opposite was true of the adults, where those with hospitalized participants were on average older (48.86 years vs 37.38 years). In both groups, a higher percentage of hospitalized participants had chronic medical conditions compared to those with non‐severe ILI. In order, the five most common chronic medical conditions in adults were as follows: cardiovascular disorder, asthma, diabetes mellitus, HIV infection, and COPD. In pediatric participants, they were congenital malformation/congenital syndrome, asthma, cardiovascular disorder, chronic pulmonary diseases, and immunodeficiencies.

**Table 1:** Baseline characteristics of all pediatric and adult participants, stratified by severe and non‐severe ILI

	Pediatric participants (<19 years)			Adults (≥19 years)		
	All Participants (N = 1530)	Severe ILI (N = 970)	Non‐Severe ILI (N = 560)	All Participants (N = 3248)	Severe ILI (N = 1083)	Non‐Severe ILI (N = 2165)
Age (SD)	3.86 (4.90)	2.44 (3.69)	6.32 (5.70)	41.21 (16.21)	48.86 (17.94)	37.38 (13.77)
Gender						
Male	825	523 (63.4%)	302 (36.6%)	1161	461 (39.7%)	700 (60.3%)
Female	705	447 (63.4%)	258 (36.6%)	2087	622 (29.8%)	1465 (70.2%)
*Z*‐score (SD)	‐0.21 (2.20)	‐0.56 (2.48)	0.38 (1.45)	27.03 (5.68)	28.05 (6.99)	26.52 (4.82)
Body mass category						
Underweight	467	382 (81.8%)	85 (18.2%)	100	55 (55.0%)	45 (45.0%)
Normal	681	377 (55.4%)	304 (44.6%)	1145	307 (26.8%)	838 (73.2%)
Overweight	204	95 (46.6%)	109 (53.4%)	1222	376 (30.8%)	846 (69.2%)
Obese	98	57 (58.2%)	41 (41.8%)	519	202 (38.9%)	317 (61.1%)
Morbidly obese	80	59 (73.8%)	21 (26.3%)	262	143 (54.6%)	119 (45.4%)
Chronic medical conditions	599	495 (82.6%)	104 (17.4%)	1229	714 (58.1%)	515 (41.9%)
ILI category						
Influenza positive	225	93 (58.7%)	132 (41.3%)	553	167 (30.2%)	386 (69.8%)
Non‐influenza virus positive	1015	702 (66.3%)	313 (30.8%)	1621	447 (27.6%)	1174 (72.4%)
Non‐influenza virus negative	290	175 (60.3%)	115 (39.7%)	1074	469 (43.7%)	605 (56.3%)

In order to determine whether body mass was correlated with severity of disease in these participants, we ran a multiple logistic regression analysis, controlling for age, sex, and presence of comorbid conditions, comparing each body mass category with normal serving as the baseline. We also included a viral pathogen grouping variable and interaction terms between BMI and viral pathogen group. The interaction terms allowed us to determine whether there were different effects in BMI for the different pathogen groups. This model showed that, in adults, the relationship between BMI and severity was significantly different for the influenza cohort (*P* < 0.0001) when compared to the virus‐negative cohort, while the relationship between BMI and severity was not significantly different in the virus‐negative and NIRV cohort. Therefore, separate logistic regression analyses were performed for the influenza cohort while the participants in the virus‐negative and NIRV cohorts were analyzed together in one larger group. In both groups, underweight and morbidly obese participants had more severe disease than normal‐weight participants. In both of those body mass categories, the effect was larger in the influenza‐positive group compared with the combined NIRV and virus‐negative group. Underweight adult participants with influenza were 5.2 (*P* < 0.005) times more likely to develop severe ILI, while their influenza‐negative counterparts had an OR of 2.88 (*P* < 0.001) (Table 2). In morbidly obese adults, these numbers were 18.4 (*P* < 0.001) and 1.89 (*P* < 0.001), respectively. While obese adults without influenza were not statistically more likely to develop severe ILI compared to normal, obese adults who were positive for influenza had an OR of 3.18 (*P* < 0.001).

**Table 2:** Odds ratio for hospitalization in participants with influenza vs other causes of ILI, based on body mass category

	Influenza positive		NIRV positive and virus negative	
	Odds ratio (N = 553)	*P*‐value	Odds ratio (N = 2695)	*P*‐value
Underweight vs normal	**5.20 (1.67, 16.01)**	**0.005**	**2.88 (1.67, 4.99)**	**<0.001**
Overweight vs normal	**1.60 (0.93, 2.78)**	**0.088**	0.90 (0.72, 1.13)	0.369
Obese vs normal	**3.18 (1.73, 5.91)**	**<0.001**	1.13 (0.85, 1.49)	0.393
Morbidly obese vs normal	**18.4 (7.83, 47.4)**	**<0.001**	**1.89 (1.34, 2.65)**	**<0.001**
Age	**1.04 (1.02, 1.05)**	**<0.001**	**1.03 (1.02, 1.04)**	**<0.001**
Sex	**0.54 (0.35, 0.84)**	**0.006**	**1.83 (1.52, 2.21)**	**<0.001**
Chronic conditions (Yes vs No)	**3.67 (2.36, 5.74)**	**<0.001**	**4.62 (3.81, 5.62)**	**<0.001**

Age, gender and presence of chronic conditions were also taken into account when running the multiple logistic regression model. Statistically significant ORs and their corresponding *P*‐values are bolded.

Because the influenza category was composed of different strains, it was of interest to determine whether differences existed between the strain of influenza and the relationship between BMI and severity. The analysis was performed on participants with only one influenza virus (A/H1N1 pdm 2009, A/H3N2, or B). No participants with other viral co‐infections were included in this strain‐specific analysis. The initial logistic regression model examined interactions between influenza strains and BMI. There was a significant interaction between BMI and influenza A/H1N1 (*P* = 0.0233) and a non‐significant interaction between BMI and influenza A/H3N2. Therefore, separate logistic regression models were performed for influenza H1N1 and a combined H3N2 and B. The risk of severe disease is increased for morbidly obese influenza A/H3N2 and B participants (OR: 5.58, *P* < 0.001, Table 3). For participants with influenza H1N1, the risk is very strong in obese (OR: 8.96, *P* < 0.001) and morbidly obese (OR: 35.13, *P* < 0.001) participants. There was also a small increase in risk of overweight influenza H1N1 participants (OR: 2.43, *P* = 0.048) compared with their normal BMI counterparts.

**Table 3:** Comparison of odds ratio for hospitalization in participants with H1N1 influenza vs H3N2 or B, and coronavirus, metapneumovirus, parainfluenza, and rhinovirus versus RSV, based on body mass category

	Influenza H1N1		Influenza H3N2 and B		Coronavirus, metapneumovirus, parainfluenza, and rhinovirus		RSV	
	Odds Ratio (N = 243)	*P*‐Value	Odds Ratio (N = 275)	*P*‐Value	Odds Ratio (N = 1403)	*P*‐Value	Odds Ratio (N = 113)	*P*‐Value
Underweight vs normal	4.62 (0.72, 24.13)	0.10	3.97 (0.61, 32.12)	0.15	**4.07 (1.71, 9.45)**	**0.002**	3.18 (0.02, 59.04)	0.536
Overweight vs normal	**2.43 (1.01, 6.46)**	**0.048**	1.20 (0.56, 2.61)	0.64	0.93 (0.67, 1.29)	0.659	2.87 (0.89, 10.06)	0.079
Obese vs normal	**8.96 (3.42, 25.87)**	**<0.001**	1.35 (0.52, 3.46)	0.53	1.28 (0.84, 1.94)	0.242	**6.33 (1.34, 35.31)**	**0.019**
Morbidly obese vs normal	**35.13 (10.43, 144.32)**	**<0.001**	**5.58 (1.46, 24.2)**	**0.011**	**2.78 (1.66, 4.65)**	**<0.001**	1.58 (0.26, 9.14)	0.606
Age	**1.05 (1.02, 1.07)**	**<0.001**	**1.03 (1.01, 1.06)**	**0.004**	**1.03 (1.02, 1.04)**	**<0.001**	**1.07 (1.04, 1.12)**	**<0.001**
Sex	**0.49 (0.26, 0.91)**	**0.024**	0.70 (0.35, 1.37)	0.292	**0.52 (0.38, 0.69)**	**<0.001**	0.68 (0.23, 1.98)	0.478
Chronic conditions (Yes vs No)	1.29 (0.63, 2.61)	0.481	**9.01 (4.69, 18.01)**	**<0.001**	**6.17 (4.61, 8.32)**	**<0.001**	**4.01 (1.46, 11.72)**	**0.007**

Age, gender, and presence of chronic conditions were also taken into account when running the multiple logistic regression model. Statistically significant ORs and their corresponding *P*‐values are bolded.

Similarly, the NIRV category was composed of different viruses and it was of interest to determine whether the BMI severity relationship was different among the most common respiratory viral pathogens present in the study. The five most common respiratory viruses were coronavirus, metapneumovirus, PIV, RSV, and rhinovirus/enterovirus. The same multiple logistic regression analysis was performed as previously described. In this analysis, only participants with a single virus were included. The interaction analysis indicated a significantly (*P* < 0.0054) different effect for the relationship between BMI and severity in the RSV group with the BMI/severity effect being similar among the other viruses. Therefore, a separate analysis was performed for the RSV group, while the participants with other viruses were combined for the analysis. In the combined group, underweight and morbidly obese participants were at higher risk of severe disease than normal‐weight participants. For the RSV participants, only obese participants (OR: 6.33, *P* = 0.019) were at higher risk than normal participants. (Table 3)

In pediatric participants, there were no significant interactions between BMI and the pathogen groups; therefore, all of the participants were analyzed together. Only obese pediatric participants were statistically significantly more likely to develop severe ILI compared to their counterparts with a normal *z*‐score (OR: 2.2, *P* = 0.002; Table 4).

**Table 4:** Odds ratio of hospitalization in pediatric participants with ILI based on body mass category

	Pediatric participants	
	Odds ratio (N = 1530)	*P*‐value
Underweight vs normal	1.21 (0.53, 2.82)	0.646
Overweight vs normal	1.15 (0.77, 1.69)	0.495
Obese vs normal	**2.20 (1.32, 3.74)**	**0.002**
Morbidly obese vs normal	1.13 (0.66, 1.94)	0.666
Age	**0.82 (0.79, 0.85)**	**<0.001**
Sex	0.99 (0.71, 1.37)	0.943
Chronic conditions (Yes vs No)	**8.73 (5.70, 13.78)**	**<0.001**

Age, gender, and presence of chronic conditions were also taken into account when running the multiple logistic regression model. Statistically significant ORs and their corresponding *P*‐values are bolded.

## 4 DISCUSSION

We have demonstrated that adults that are underweight or morbidly obese are more likely to be hospitalized from an influenza‐like illness, regardless of the causative agent of the illness, than normal‐weight adults. The risk follows a “U”‐shaped curve, where individuals at both extremes (ie, those that are underweight or morbidly obese) were more likely to develop severe ILI when compared with normal‐weight, overweight, or obese individuals. The increased risk of underweight and morbidly obese adults was stronger for influenza‐positive adults compared with those positive for other respiratory viruses or negative for any respiratory viruses.

Analysis of the risk of specific respiratory viral pathogens or strains of influenza was difficult given the small number of participants in each of these groups. Despite the large confidence intervals, the data seem to indicate that the association between morbid obesity and risk of severe ILI is not just statistically significant in adults with influenza A/H1N1 pdm 2009. A statistically significant OR was also found in participants with influenza H3N2 and B. While the association was also present for these strains of influenza, the odds ratio was six times higher in influenza H1N1 participants (OR: 35.13 vs 5.58), which may explain why individuals with H1N1 were the first in which the association between body mass and disease severity was postulated. However, influenza H3N2 and influenza B still have twice the risk of hospitalization as the other most common respiratory viruses. Preclinical data suggest reduced vaccine efficacy in obesity,^24^, ^25^ and while the increased risk does not seem to be due to reduced antibody titers,^26^, ^27^ it may be from reduced cellular immunity.^28^ However, this would not explain the differential risk of H1N1 over H3N2, nor of all influenza over other respiratory viruses.

The risk associated with low body mass is a finding that has previously been described as it relates to all‐cause mortality,^29^, ^30^ but not to the severity of influenza‐like illness. Being underweight seems to be a consistent risk factor in all adult participants with ILI.

Our analysis did not demonstrate a clear association between body mass and the risk of severe ILI in children. A few prior studies found obesity to be a risk factor.^31^, ^32^ Recent studies suggest BMI *z*‐scores, as used for pediatric participants in this study, are only weakly associated with true adiposity.^33^ Therefore, our categorization may not reflect the appropriate categories relevant to categorizing immune dysfunction from obesity.

One significant limitation to our study is that it enrolled participants that sought medical care for an influenza‐like illness. It is not a population‐based study that can accurately assess the risk that body mass confers for infection. Additionally, the study cannot differentiate within the NIRV group between those with true infections (but not detected on the multiplex platform), or those with non‐infectious etiologies of the respiratory symptoms.

In conclusion, our findings suggest that adults, who are underweight or morbidly obese, even if they do not have chronic conditions that increase the risk of influenza‐related complications, may be at increased risk of developing severe disease due to seasonal influenza infection as well as other respiratory viral infections. Clinicians should keep a patient's body mass index in mind when evaluating risk and deciding on a course of treatment.

## CONFLICT OF INTEREST

The authors have no conflict of interest to declare.
